# Evaluation of two different self-disinfection alginate impression material

**DOI:** 10.1038/s41405-024-00269-6

**Published:** 2024-11-05

**Authors:** Islam M. Bendary, Alaa A. Omar, Reham M. Goda, Ahmed A. Ali, Kareem A. Lotfy, Mohamed M. Shohayeb

**Affiliations:** 1 Department of Conservative Dentistry, Faculty of Dentistry, Delta University for Science and Technology, Gamasa, 11152 Dakahlia, Egypt; 2 Nanomedicine Research Unit, Delta University for Science and Technology, Gamasa, 11152 Dakahlia, Egypt; 3 Department of Microbiology and Immunology, Faculty of Pharmacy, Delta University for Science and Technology, Gamasa, 11152 Dakahlia, Egypt; 4 Department of Prosthodontics, Faculty of Dentistry, Delta University for Science and Technology, Gamasa, 11152 Dakahlia, Egypt; 5 Department of Conservative Dentistry, Faculty of Dentistry, Deraya University, Minya, Egypt

**Keywords:** Infection control in dentistry, Dental materials

## Abstract

**Objective:**

This study was conducted to evaluate the antimicrobial efficacy and mechanical properties of two experimental self-disinfecting alginate preparations utilising two different antimicrobial agents; povidone-iodine and silver nanoparticles.

**Methods:**

Alginate moulds were assigned into three groups. Additives-free control group, povidone-iodine-containing group, and silver nanoparticle-containing group. Discs from each group were obtained and assessed for their antimicrobial activities by the disc diffusion method against *Staphylococcus aureus*, *Escherichia coli*, and Candida albicans. For the elastic recovery analysis, each group was divided into 6 samples. Each sample was mixed according to its group specification and subjected to surface detail reproduction and elastic recovery according to ISO 1563:1990 specifications. All data were expressed as mean ± standard deviation for each group at the significance level of *P* < 0.05.

**Results:**

Results revealed that the experimental self-disinfection alginate possessed broad-spectrum antimicrobial activities against the tested microorganisms, compared to the control group. No statistically significant differences in elastic recovery values between all tested groups (*P* < 0.05) were observed. For surface detail reproduction, all samples reproduced the 50 μm line.

**Conclusion:**

Povidone-iodine as well as silver nanoparticles could be used efficiently for the sanitization of alginate moulds without adverse effects on detail accuracy or elastic recovery of the impression material.

## Introduction

Initial impressions are one of the routine procedures in dental clinic practices before starting treatment. Successful impression tacking is a main concern in achieving an accurate gypsum model as a primary or diagnostic cast. Tracking this point, accuracy and dimensional changes are critical factors that should be considered while the impression is tacked and poured [1]. Digital impressions could be considered an appropriate method for recording oral cavity details without the hazards of conventional methods [2]. Despite these vantages, conventional impressions are still widely affordable for recording oral cavity fine details [3]. Usually, mould accuracy is greatly influenced by environmental factors, time of setting, water content and the composition of the impression material [4].

One of the most common, reliable and affordable materials for primary impressions is alginate. Alginate was introduced as a widely available material that could duplicate all the fine details in the oral cavity [5–7]. After setting and removing the impression mould from the oral cavity, the impression should be washed with water spray and disinfected with an antibacterial solution [8]. Since alginate is naturally hydrophilic, at this point, distortion due to these aqua disinfectants could cause expansion and dimensional changes up to 0.1% [9]. Changes that could result from this distortion probably, have an impact on the quality of the final cast accuracy and ultimately the success of the final restoration [10].

Cross-infection protocols as per the American Dental Association (ADA) requirements obligate the operator to disinfect dental impressions before using [8–10]. Impressions should be free from contamination with pathogenic microorganisms from the patient’s fluids, as this could present a significant cross-infection risk for harmful microorganisms [11]. Researchers introduced many different chemicals as disinfection solutions, but, dimensional instability is still a persisting problem [12].

To address this problem, disinfectants were incorporated into the composition of the irreversible hydrocolloids to become a “self-disinfectant” [13–15]. In this study, the well-known antimicrobials; silver nanoparticles (AgNPs) and Povidone-iodine (PV-I) were compared for their disinfecting efficiency. Their self-disinfection property was tested against *Staphylococcus aureus* (Gram-positive bacterium), *Escherichia coli* (Gram-negative bacterium) and *Candida albicans* (yeast).

The null hypothesis to be tested is that adding PV-I and AgNPs could adversely affect mechanical properties and the accuracy of the experimental alginate impressions.

## Materials and methods

This study protocol has been approved by the Research Ethics Committee at the Faculty of Pharmacy, Delta University for Science and Technology (FPDU-REC) and holds the approval number FPDU7/2024. This approval ensures that this research has been planned, conducted, documented, and reported according to regulatory requirements and various ethical guidelines.

### Chemicals and media

Materials used in the study were commercial alginate impression material (IQ green, Lascod, Italy, Batch no. 0159311) and povidone-iodine (PV-I, Merck, Darmstadt, Germany). While, chemicals used for AgNPs synthesis: polyvinyl pyrilidone (PVP, MW ~ 40,000, Oxford), silver nitrate (AgNO_3_, Piochem, Giza, Egypt), sodium hydroxide (NaOH, Alpha Chemika, India), sodium carbonate (Na_2_CO_3_, Alpha Chemika, India), acetone HPLC grade (Merck, Darmstadt, Germany) and glucose (Alpha Chemika, India). For antibacterial and antifungal tests Mueller-Hinton and Sabouraud dextrose agar (Oxoid, UK) were used respectively.

### Bacterial strains

Clinical culture isolates of *Staphylococcus aureus* (a gram-positive bacteria), *Escherichia coli* (a gram-negative bacteria) and *Candida albicans* (a yeast) were procured from Microbiology and Immunology Department, Faculty of Pharmacy, Delta University for Science and Technology.

### Methods

#### Preparation and characterization of silver nanoparticles (AgNPs)

AgNPs were prepared by chemical reduction of AgNO_3_ (0.01 M) with glucose in the presence of PVP (1%) in a water bath at 55 °C for 1 h with vigorous stirring. The pH was kept at 8.5–9.0 using NaOH and Na_2_CO_3_ to enhance the reaction velocity. AgNPs were precipitated by washing several times with acetone, separated using centrifugation at 6000 rpm for 10 min, and subsequently allowed to air dry in a desiccator [16]. The dried AgNPs were re-dispersed in deionized water using an ultrasonic bath for characterizing the maximum absorption spectra using UV-Vis spectrophotometer (UV-900I, Shimadzu, Japan). Particles size and distribution were estimated by a transmission electron microscope (TEM, JEOLJEM 2100, JOEL ltd, TYO, Japan).

### Preparation of the self-disinfecting dental alginate

A weight of 0.45 g of dental alginate powder was mixed for 45 s with either 1 mL of water (control), 1 mL of 10% PV-I (PV-I-alginate) or 1 mL of 0.05% AgNPs suspension (AgNPs-alginate). The pastes were spread on an even surface to a thickness of 0.5 mm. Discs of 5 mm diameter were cut using a cork borer, and stored in a sterile Petri dish till used within an hour.

### The antimicrobial activity of the self-disinfected dental alginate

The antimicrobial activity of dental alginate containing PV-I and Ag-NPs was assessed by the disk diffusion method. Log-phase bacterial cultures of *S. aureus, E. coli* and *C. albicans* and adjusted to 10^5^ CFU/mL with the help of a standard 0.5 McFarland. Hundred µL of each culture was spread on the surface of sterile Mueller-Hinton agar plates, in the case of bacteria and Sabouraud Dextrose agar, in the case of *C. albicans* and left for 10 min. The discs were loaded on the surfaces of the agar plates and incubated for 18 h at 37^o^C for bacteria and for 4 days at 28^o^C for *C. albicans*. The diameters of the obtained zones of inhibition around each disc were measured. The experiment was repeated three times and means of inhibition zones were calculated.

### Surface details reproduction test

**Fig. 1:**
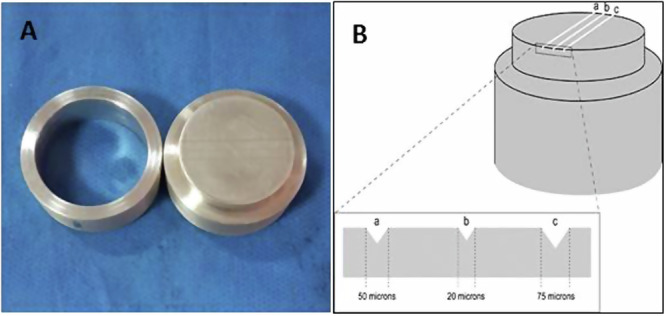
The surface details reproduction test was done according to ISO specification 1563. **A** Cupper mold; **B** a diagram for the three vertical orientation lines spaced 2.5 mm apart. The lines are 50, 20, and 75 µm depth and 25 mm length.

Details reproduction test was performed according to ISO specification 156312 [17]. Six samples from each treated alginate group were analysed and compared to the control group. In each group, a copper mold engraved with three parallel lines spaced 2.5 mm apart were used. The lines had 50, 20, and 75 μm width and all had 25 mm in length. Two supplementary lines denoted X and X’ were employed for the assessment and replication of the three lines surface details (Fig. 1A, B). Each sample was prepared individually according to method of preparation and poured into the ring-matrix assembly. The rigid mold plate was then subjected over the assembly, to ensure a tight sealing. The material was subjected to a constant weight of 2 kg during setting to simulate forces applied during impression tacking process and allowed for excess material escape. The assembly was immersed in a water bath at 35 °C to mimic the temperature inside the oral cavity and left for the recommended time for setting. After complete setting, the blocks were taken out of the water bath and the ring mold was separated from the test blocks. The surface of each specimen was examined after gelation, according to the ISO specifications. The alginate tested blocks were scored on a scale from zero to one as per the ability of the material to replicate the full length of a 50 µm line.

### Elastic recovery test

**Fig. 2:**
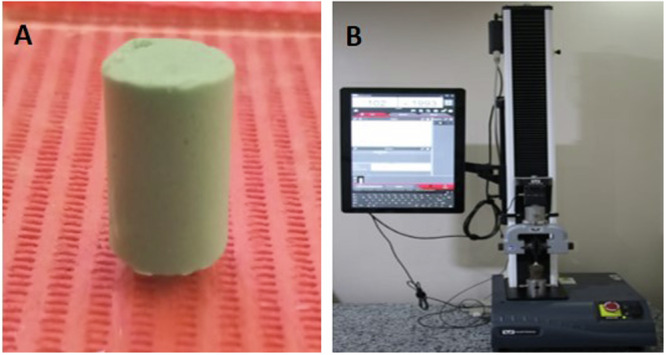
Elastic recovery assessment accordance to ISO 1563:1990. **A** Split cylindrical mold (20 mm in length and a 12.5 mm interior diameter). **B** Testing machine.

Elastic recovery assessment was conducted in accordance with ISO 1563:1990 specifications. A segmented cylindrical mold with dimensions of 20 mm in length and an interior diameter of 12.5 mm, encased within a securing ring (Fig. 2A) was utilised. Each sample (*n* = 6) of the three groups was prepared, as described above, allowed to be set entire the mold and then inspected for standardization. Test performed using material testing machine (Zwick Zmart Pro model, produced by Zwick Roell GmbH & Co. KG in Ulm, Germany) (Fig. 2B). Twenty percent of the original length (L) was gradually deformed for 5 seconds, and then released to facilitate recovery with a period of 40 seconds, the samples underwent measurement once more. The extent of recovery from deformation was quantified in terms of percentage using the subsequent equation:

Elastic recovery = (ΔL/L − 1) × 100 where L is the original length and ΔL represents the length after deformation.

### Statistical analysis

Experimental Data were statistically analysed using SPSS Software (version 20.0). The significance value *p* ≤ 0.05 was applied. Data distribution was examined using the Shapiro–Wilk test. The elastic recovery data were analysed via Duncan’s multiple range and Tukey post-hoc test for group comparison, when the ANOVA test was significant.

## Results

### Characterization of AgNPs

**Fig. 3:**
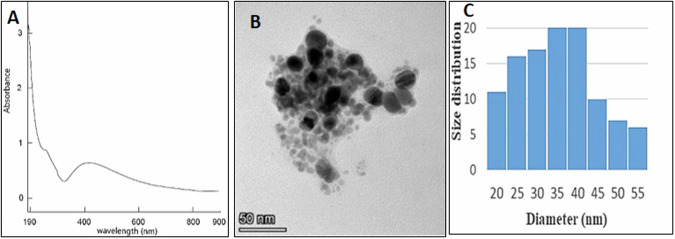
Characterization of AgNPs. UV spectrum (**A**), TEM micrograph (**B**) and particle size distribution (**C**) of AgNPs.

The absorption spectrum of AgNPs illustrated in Fig. 3A, showed a strong broad peak. The formation and the characteristics of AgNPs described a well-defined single plasmon band at 420 nm. Moreover, the broadening of the peak signified the wide dispersion of the particles. Also, the TEM micrograph portrayed in Fig. 3B exhibited a well-dispersed spherical shape of nanosized Ag+. Figure 3C showed a particle size distribution ranging between 20 and 55 nm, with an average of 38.6 ± 0.3.

### Antimicrobial activity of the self-disinfected dental alginate

**Fig. 4:**
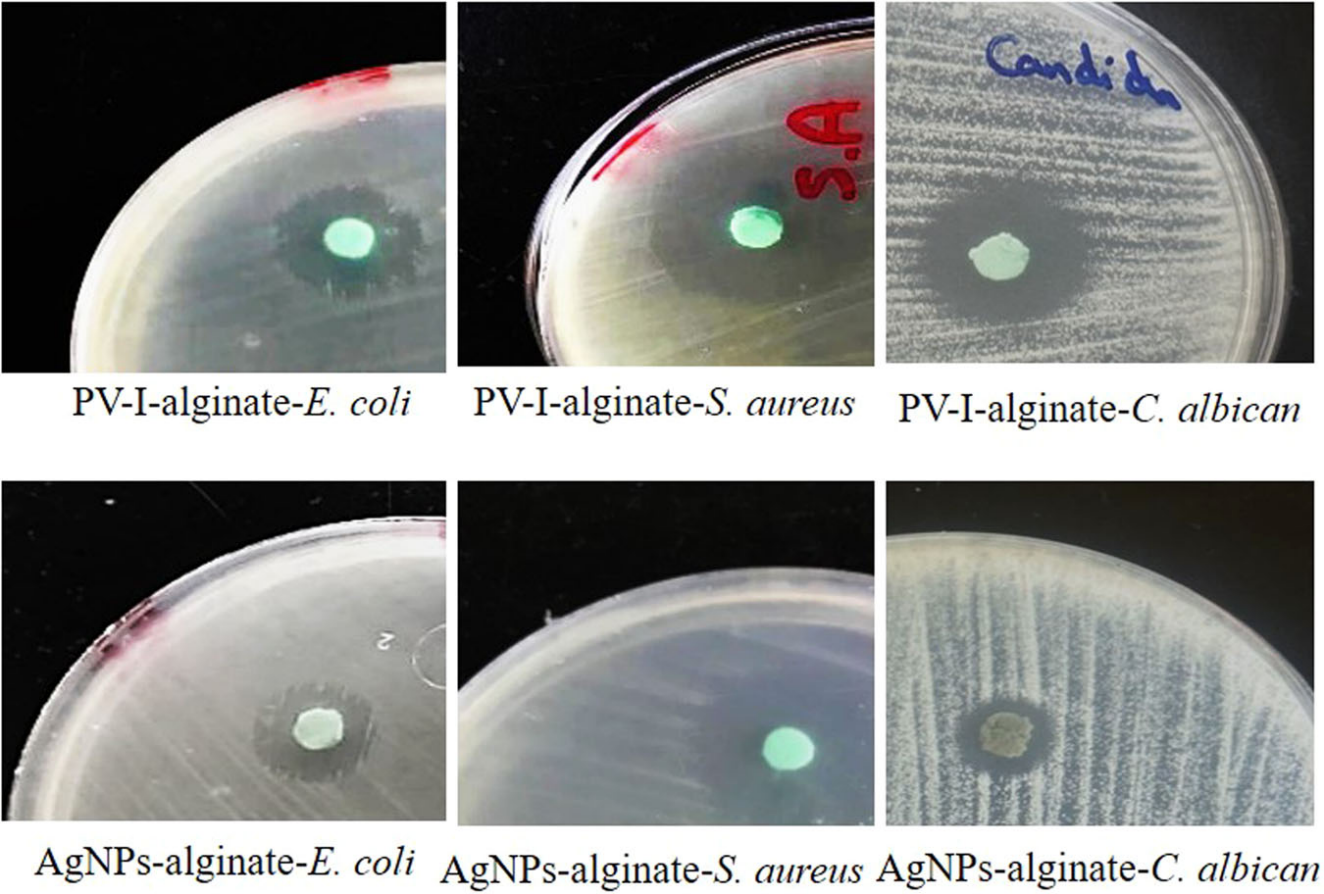
Evaluation of the antimicrobial activity of alginate containing AgNPs and PV-I as disinfectants by disc diffusion method.

Disc diffusion method revealed that both AgNPs-alginate and PV-I-alginate exhibited a broad–spectrum activity against the three tested organisms and significantly (*p* ≤ 0.05) more active against the *S. aureus* (G+) than *E. coli* (G-) (Table 1, Fig. 4). PV-I-alginate was significantly more active against *C. albicans* than AgNPs-alginate.

**Table 1:** Inhibition of *Staphylococcus aureus, Escherichia coli* and *Candida albicans* by AgNPs-alginate and PV-I-alginate discs.

Disinfectant-alginate	Diameter (mm)		
	*S. aureus*	*E. coli*	*C. albicans*
AgNPs-alginate	20.0 ± 2.0	13.0 ± 1.0	11.0 ± 1.5
PV-I-alginate	22.5 ± 3.0	15.0 ± 2.0	21.0 ± 2.5

### Surface details reproduction and elastic recovery of the self-disinfected dental alginate

**Fig. 5:**
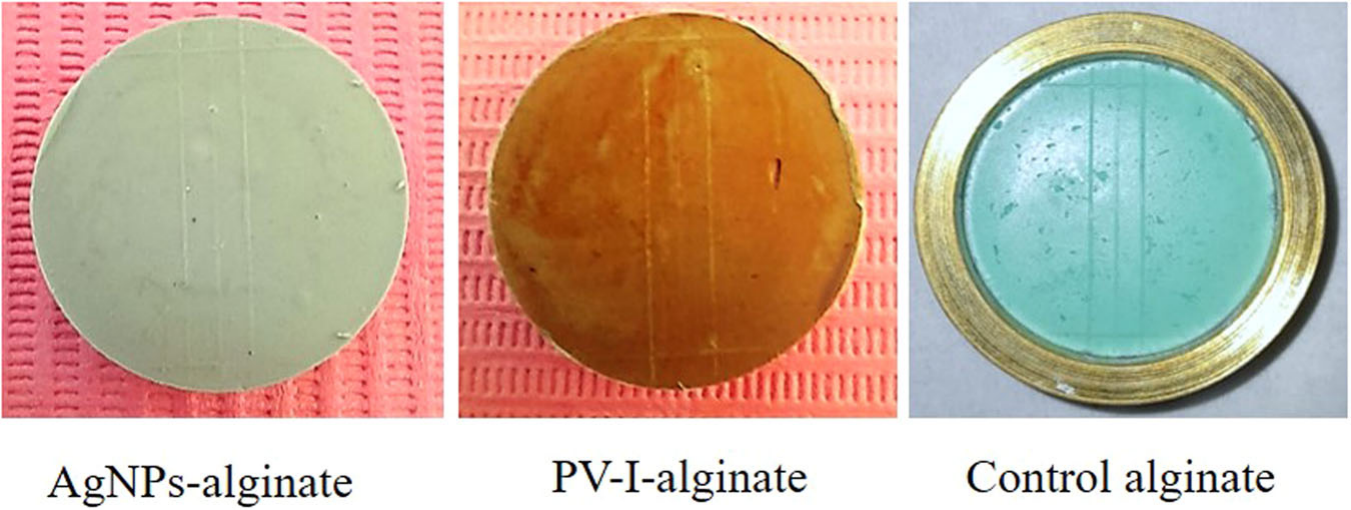
Surface details reproduction of all alginate impression materials samples completely reproduced on the 50 μm-line.

The surface details reproduction of all alginate samples complied with the reproduction test ISO specification 1563. The samples completely reproduced the 50μm-line regardless of the disinfection procedure used which in return recorded a score of one as shown in (Fig. 5).

**Fig. 6:**
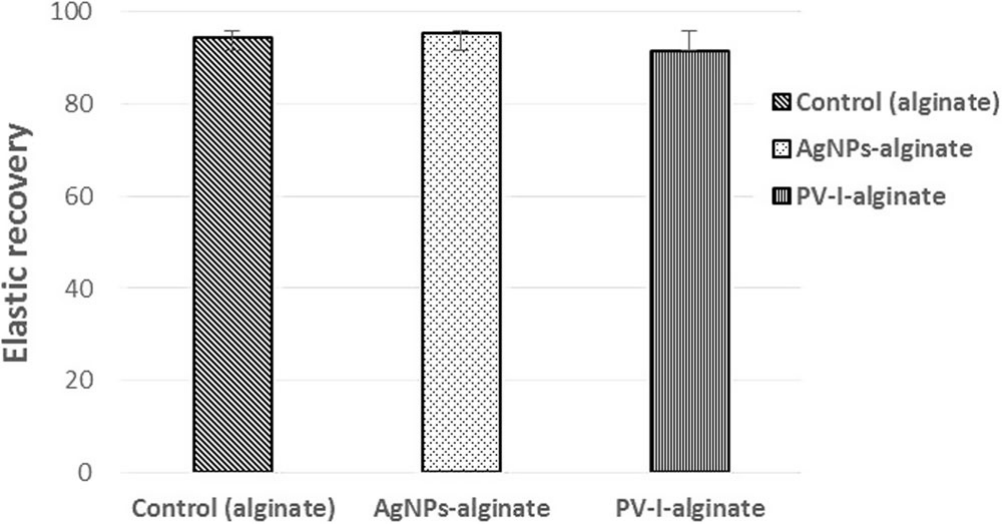
Elastic recovery of AgNPs-alginate and PV-I-alginate compared to the control.

The elastic recovery test values of the control and AgNPs-alginate samples were close to the minimum range described in ANSI/ADA specification no. 18-1992 which is a 95% recovery. While there was no statistically significant difference in the mean values between the control, PV-I-alginate and AgNPs-alginate groups (*p* > 0.05), whereby the scores were 94.36 ± 0.26, 91.53 ± 0.47 and 95.55 ± 0.34 respectively (Fig. 6).

## Discussion

This study was held to focus on the influence of using self-disinfection alginate impression material instead of conventional methods of disinfection including sprays or immersion techniques that could adversely affect proper accuracy. Elastic Impression materials should have minimal rheological changes to record dental and bony undercuts in the oral cavity. The minimum range of elastic recovery proposed by ANSI/ADA specification no. 18-1992 was 95% [18]. The addition of betadine negatively relatively reduced the elastic recovery to 91.53 ± 0.47 compared to the ANSI/ADA specification. However, this reduction, compared to the control, was statistically insignificant. In addition, results obtained in the present study suggest that the mechanical properties of the alginate impression were not altered. Therefore, the null hypothesis could be rejected.

To avoid the possibility that the acidic pH of betadine may irritate oral tissues, the pH of the alginate PV-I mixture was checked and found to be 6.8 (data not shown). This suggests that alginate buffered the pH of the final preparation to nearly a neutral pH.

Bacterial colony growth in the control group confirmed the ability of oral microorganisms to attack dental impressions, and the inability of dental alginate to perform a self-disinfection property [19]. The hydrophilic nature and the porous structure of dental alginate proposed a proper field for both bacterial growth and the accessibility of water imbibition [20]. Disinfection could be achieved by adding nanofiller additives with proper concentration [15]. AgNPs have been used as a disinfectant agent for various biomedical applications [21] due to their nontoxicity at low concentrations [22, 23]. The antimicrobial and antiviral effect of AgNPs is ascribed to the capacity of the released Ag+ions, which possess a positive charge to engage with the negatively charged membranes of the microbial cells [24]. The particle size of the synthesised AgNPs is a key factor in the antimicrobial activity. The size of AgNPs exhibited high antimicrobial activity because of the abundance of the Ag+ released from AgNPs. The obtained size enabled particles to attack bacterial surfaces and lysis of their cell wall [25], which was confirmed by disk diffusion tests.

AgNPs and PV-I incorporated into dental alginate provided effective disinfection without alteration of the mechanical properties of the material. PV-I as a disinfectant was tested and evaluated as effective [26]. PV-I has a well-established broad-spectrum antimicrobial activity against Gram-positive, Gram-negative, mycobacteria and yeast [27, 28]. In addition, it has a wide range of activity against viruses [29, 30]. The microbicidal activity of the iodine released from PV-I is reported via the deactivation of vital bacterial cellular mechanisms and structures. Also, it may cause nucleotides and fatty/amino acids oxidation [31]. In addition, it inactivates the membrane respiratory chain enzymes of bacteria [32]. The bactericidal effect is exerted within 15 s, without harming human cells [33].

Antimicrobial results demonstrated that after incorporating AgNPs and PV-I in alginate, they exhibited a broad spectrum of antimicrobial activity on *S. aureus, E. coli* and *C. albicans*. Both were significantly more active against Gram-positive bacteria than Gram-negative bacteria (*p* ≤ 0.05), as previously reported [26, 34]. Data obtained reveal that PV-I alginate was highly active against *C. albicans*, which agrees with a previous study [35]. The lethal effect at concentrations between 0.05 and 10% has been reported [36].

Diluted PV-I is lethal to periodontal pathogens like *Aggregatibacter actinomycetemcomitans, Porphyromonas gingivalis*, *Klebsiella pneumoniae* and *Streptococcus pneumoniae* [37, 38]. PV-I also kills different viruses like, herpes viruses [38], SARS-CoV, influenza virus A (H1N1) and rotavirus [32], after 15 s with no or rare allergic sensitisation. Therefore, it is widely used as a subgingival irrigant [39].

Specifications provided by the ADA document number 18 [15, 40, 41] and the guidelines established by the International Organization for Standardization in reference 1563 pertain specifically to the use of dental alginate. These documents stipulated precise demands and restricted dimensional change values. The results of the current study met these specifications with no significant statistical differences between all tested groups in terms of details reproduction accuracy and elastic recovery.

ISO standards of details reproduction test were applied successfully in all the tested specimens as they recorded the 50 µm line entire length. This suggests that the concentrations of the tested agents used in the study did not alter the impression flow and hence, a proper details reproduction was obtained [15]. Data obtained suggest that the antimicrobial additives homogenised within the alginate matrix were inert and did not affect the intermolecular bonds of the formed alginate gel.

The presence of Ag-NPs at a concentration below 1.0% was reported to strengthen the formed cross-linked alginic acid chains [25]. This may be due to its influence on the rheological properties as demonstrated in the elastic recovery test. On the other hand, there was no significant decrease in the elastic recovery of alginate after PV-I addition. The reduction could be due to a decrease in water available in the presence of the highly water-soluble PV-I that resulted in a lower elastic recovery [18].

## Conclusion

Data obtained, indicated that the incorporation of both PV-I and AgNPs exhibited a broad spectrum antimicrobial activity. They exerted no adverse effects on the surface details reproduction of alginate. Furthermore, there was no significant effect on the elastic recovery of the alginate impression material. Although both PV-I and AgNPs could be used as efficient disinfectants for alginate impressions. It seems reasonable to recommend PV-I for alginate disinfection because it is cheaper and more easily accessible.

## Data Availability

The original contributions presented in the study are included in the article, further inquiries can be directed to the corresponding author/s.
